# Molecular Cloning, In Silico Characterization, and Promoter Analysis of a Putative *ETHE1* Gene from *Jatropha curcas*

**DOI:** 10.3390/cimb48090919

**Published:** 2026-09-08

**Authors:** Mei-Li Zhao, Feng-Jin Han, Lei Nie

**Affiliations:** College of Life Sciences, Xinyang Normal University (XYNU), Xinyang 464000, China; han.fengjin@outlook.com

**Keywords:** *Jatropha curcas*, *ETHE1*, sulfur dioxygenase, bioinformatics analysis, phylogenetic analysis

## Abstract

Sulfur metabolism and stress responses are fundamental to plant growth and seed development. ETHE1 encodes a mitochondrial sulfur dioxygenase that plays important roles in these processes; however, its function in *Jatropha curcas* remains unknown. In this study, we report the cloning and in silico characterization of a putative *JcETHE1* gene from *J. curcas*. Bioinformatic analysis revealed that *JcETHE1* has an open reading frame of 726 bp encoding a 241-amino-acid protein, which is predicted to localize in mitochondria and possesses a typical sulfur dioxygenase conserved domain. Because the transcript ends were not experimentally confirmed and the predicted protein is shorter than related plant homologues, the possibility that the cloned sequence is partial cannot be excluded. Phylogenetic analysis showed that JcETHE1 clusters within the Euphorbiaceae clade, showing high similarity to ETHE1 from *Manihot esculenta* and *Hevea brasiliensis*. In silico promoter analysis suggested that, in addition to core elements, the *JcETHE1* promoter contains multiple putative regulatory elements associated with light response, hormone responses (ABA, ethylene, gibberellin), floral development (CArG-box, AAGAA-motif), stress responses, and endosperm development. These findings provide a molecular basis for future functional studies of JcETHE1 and suggest that it may serve as a potential candidate gene for further investigation into reproductive development and stress responses in *J. curcas*. However, these hypotheses require experimental validation.

## 1. Introduction

*Jatropha curcas* L., a member of the Euphorbiaceae family, is an energy plant known for its high seed oil content, making it an important feedstock for biodiesel [1,2]. Moreover, the fatty acid composition of its seed oil is similar to that of edible oils. However, the presence of toxic phorbol esters has historically restricted its use as a food source, and extensive detoxification processes are required prior to any potential application in this regard [3]. *J. curcas* produces female and male flowers in the same inflorescence, but its high male-to-female flower ratio significantly limits its seed productivity [4,5]. In addition, *J. curcas* is well established in most tropical and subtropical regions of the world, where it is often cultivated on marginal lands such as poor, arid, and high-altitude areas [6]. It frequently encounters various adverse conditions, including low temperature, drought, salinity, heavy metal stress, and others, which significantly impact its yield and oil quality [7]. Therefore, identifying key genes involved in flower development and stress responses is crucial for genetic improvement programs aimed at enhancing yield and stress tolerance in this species.

ETHE1 (Ethylmalonic encephalopathy protein 1) encodes a mitochondrial sulfur dioxygenase belonging to the metallo-β-lactamase superfamily [8]. It catalyzes the oxidation of glutathione persulfide (GSSH) to sulfite, thereby completing sulfide detoxification [9,10]. In humans, ETHE1 mutations cause ethylmalonic encephalopathy [11].

Functional studies of ETHE1 in plants have primarily focused on *Arabidopsis thaliana*, rice, and grapevine. AtETHE1 is essential for embryo development and high protein turnover, and it participates in the utilization of amino acids as alternative respiratory substrates [12,13]. Knocking out the *AtETHE1* gene leads to abnormal endosperm development, delayed embryonic development, and ultimately embryo death [12]. In the knockdown mutant *ethe1*, sulfur dioxygenase activity decreases to 1%; although embryos remain viable, their development is severely delayed [13,14]. Furthermore, *AtETHE1* is up-regulated under carbohydrate starvation (such as prolonged darkness) and stress conditions (drought, abscisic acid treatment), indicating a potential role in plant responses to abiotic stress [13,14].

*OsETHE1* is specifically expressed in roots and is induced by multiple abiotic stresses, including heat, drought, and salt, thereby affecting plant nutritional status [8,15]. In grapevine, *VvETHE1A* responds to ABA and is hypothesized to regulate endosperm development through the ABA pathway, whereas *VvETHE1B* strongly responds to methyl jasmonate (MeJA), suggesting its involvement in the MeJA signaling pathway [10].

*AtETHE1* exhibits high expression in callus, inflorescences, and siliques, and is critical for seed production and embryo development [12]. Immunolocalization studies demonstrated that AtETHE1 signals are most intense in the anther chamber vegetative cells (e.g., tapetal cells) of flower buds and are present in the embryo and endosperm during early seed development [12]. In grapevine, *VvETHE1A* is highly expressed in stems, leaves, flower buds, tendrils, and embryos, while *VvETHE1B* is strongly expressed in roots [10]. These studies reveal expression differences and functional diversity of *ETHE1* across different species.

In this study, we report the cloning and in silico characterization of a putative *JcETHE1* gene from *J. curcas*. Our results provide a molecular basis for further functional dissection of *JcETHE1* and suggest that it may serve as a candidate gene for future investigation into reproductive development and stress responses in this energy crop, pending experimental validation.

## 2. Materials and Methods

### 2.1. Plant Materials

*J. curcas* plants used in this study were cultivated at Guangxi University. Fresh young leaves of mature *J. curcas* were collected, separated and frozen immediately in liquid nitrogen, and stored at −80 °C.

### 2.2. Cloning of JcETHE1

Total RNA was extracted from the young leaves of *J. curcas* using a Biozol (Biom, Hangzhou, China) RNA extraction kit according to the manufacturer’s instructions. The quality and concentration of the RNA were determined with a NanoDrop 2000 spectrophotometer (Wilmington, DE, USA). First-strand cDNA was synthesized using the PrimeScript™ RT Reagent Kit with gDNA Eraser (TaKaRa, Dalian, China). The nucleotide sequence of *JcETHE1* was obtained from genome sequencing data and used as a template to design cloning primers (GCTAAGTGCTGCCCGATTTC and GCATGATCAGCTGGACAAATCA) for amplifying the CDS of *JcETHE1*. The cloned cDNA sequence has been deposited in the NCBI GenBank database under the nucleotide accession number PZ567662.

### 2.3. Bioinformatics Analyses of ETHE1 Homologues

For the identification of the *ETHE1* gene in the *J. curcas* (*Jatropha curcas* genome assembly RJC1_Hi-C—NCBI-NLM (nih.gov)) [16], the cloned CDS of the *JcETHE1* gene obtained from the study was used as a query to the BLASTn (version 2.17.0) algorithm, with an E-value cut-off threshold of 1 × 10^−10^. The BLASTn search identified a genomic scaffold (GenBank accession no. NW_023591682.1) showing the highest similarity to the cloned sequence. This search yielded a query coverage of 100%, a percentage identity of 98.68%, an alignment length of 726 bp, and an E-value of 0.0. Subsequently, the corresponding genomic locus on the *J. curcas* genome assembly scaffold (GenBank accession no. NW_023591682.1) was identified, which is located on the reverse (complement) strand at positions 1,343,323–1,354,051 (GenBank Assembly ID: GCF_014843425.1). Since the exact transcription start site (TSS) has not been experimentally validated, a conservative 2000-bp sequence immediately upstream of the translation initiation codon (ATG) from this genomic scaffold was extracted and used as a putative promoter region. This promoter sequence is provided in Supplementary Table S1. This sequence information was used for phylogenetic relationship, gene structure and protein motif analyses. Conserved domains and the protein superfamily were predicted using InterPro (version 109.0, 6 March 2026, https://www.ebi.ac.uk/interpro/) [17]. The physicochemical properties of *JcETHE1* (including molecular mass, isoelectric point, and amino acid composition) were analyzed with the ProtParam tool on the ExPASy server (6 March 2026, https://web.expasy.org/protparam/) [18]. Subcellular localization of the ETHE1 proteins was predicted using the Plant-mPLoc online tool on the Cell-PLoc-2 website (6 March 2026, http://www.csbio.sjtu.edu.cn/bioinf/plant-multi/) [19]. Secondary and tertiary structures of JcETHE1 were predicted using SOPMA (6 March 2026, https://npsa.lyon.inserm.fr/cgi-bin/npsa_automat.pl?page=/NPSA/npsa_sopma.html) [20] and the Phyre2 Protein Fold Recognition Server (6 March 2026, https://www.sbg.bio.ic.ac.uk/phyre2/html/page.cgi?id=index) [21,22], respectively. Conserved motif analysis of JcETHE1 and its homologous proteins (from *Arabidopsis*, rice, grapevine, and humans) was performed with MEME 5.5.9 (MEME Suite; [23]), with the parameters set to five motifs and a width of 6–50 amino acids. The obtained motifs were searched and validated using MAST [24] with an E-value threshold of 10 and a position *p*-value threshold of 0.0001. A 2000 bp sequence upstream of the *JcETHE1* start codon was used as the promoter region and submitted to PlantCARE (6 March 2026, http://bioinformatics.psb.ugent.be/webtools/plantcare/html/ [25] and PlantPAN 4.0 (6 March 2026, https://plantpan.itps.ncku.edu.tw/) [26] for prediction of *cis*-regulatory elements. The authenticity of this promoter region was manually verified against the *J. curcas* genome assembly (RJC1_Hi-C). According to the NCBI genomic annotations, we confirmed the gene orientation, the absence of overlapping CDSs from neighboring genes within this 2000-bp window, and the annotated 5′ UTR information. To avoid redundant counting, overlapping or duplicated predictions were handled according to the following criteria: (1) identical elements predicted by both databases were recorded only once, with priority given to the PlantPAN annotation; (2) when different motifs overlapped on the same strand, the one with the longer sequence length was retained; (3) redundant elements with identical names and start positions were merged. The promoter sequence was analyzed using the web-based versions of PlantCARE and PlantPAN 4.0 with their default search parameters. In these databases, a motif is reported when the sequence matches the consensus matrix of the database with the default similarity score (typically >80% similarity to the core consensus sequence), as implemented by the respective platforms. No additional manual threshold was applied. The distribution of elements was visualized using TBtools (version 2472).

### 2.4. Phylogenetic Analysis

The accession numbers of the amino acid sequences of ETHE1 (Supplementary Table S2) from Euphorbiaceae, Salicaceae, Solanaceae, Brassicaceae, Poaceae species, and humans were retrieved from the NCBI non-redundant protein database and TAIR (6 March 2026, https://www.arabidopsis.org/) [27] via BLASTp searches using the JcETHE1 sequence as a query, with an E-value cutoff of <1 × 10^−10^ and sequence identity > 40%. Multiple sequence alignment was performed using the PPP algorithm of MUSCLE v5.3 [28] with default parameters. The final alignment comprised 334 amino acid positions, including all gap-containing columns (gaps were treated as missing data). The alignment file is provided in Supplementary File S1. The optimal substitution model was selected using ModelFinder [29] based on the Bayesian Information Criterion (BIC). Maximum-likelihood (ML) phylogenetic analysis was conducted using IQ-TREE v3.0.1 [30] with the Q.plant + G4 model, which was identified as the best-fit model. Branch support was assessed using 10,000 ultrafast bootstrap replicates [31] with a bootstrap correlation coefficient of 0.90. The phylogenetic tree file (Newick format) is provided in Supplementary File S2. The tree was rooted using human ETHE1 sequences as an outgroup. For phylogenetic analysis, multiple isoforms from the same species were intentionally retained in the dataset. This decision was made not only to explore interspecific relationships but also to provide an overview of the intraspecific sequence heterogeneity, despite the potential trade-off of lowering bootstrap support at certain internal nodes.

## 3. Results

### 3.1. Cloning and Sequence Analysis of JcETHE1

**Figure 1:**
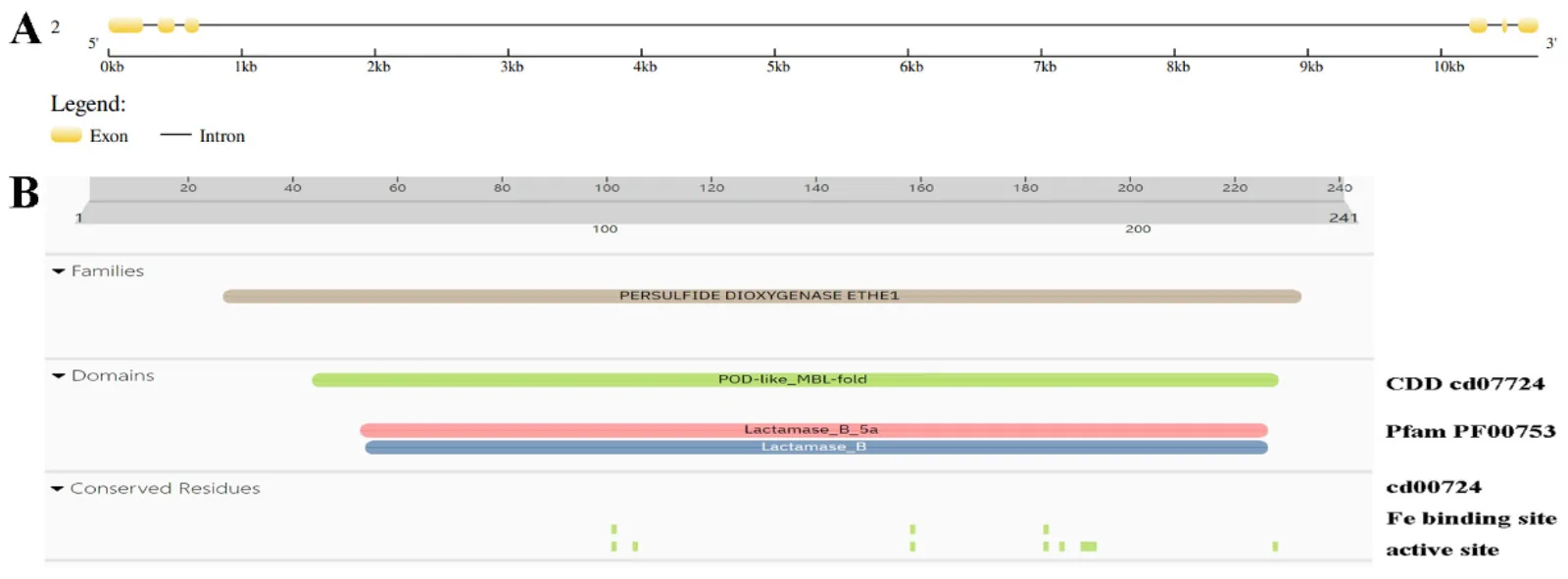
Gene structure of *JcETHE1* (**A**) and conserved domain and superfamily analysis of the JcETHE1 protein (**B**). Note: The nucleotide sequence reported in this study is available under GenBank accession no. PZ567662. The corresponding protein accession number will be automatically assigned by NCBI upon final processing of the submission.

The genomic sequence of the *JcETHE1* gene is 10,729 bp in length and comprises six exons (GenBank accession no. NW_023591682.1) (Figure 1A). The *JcETHE1* cDNA (GenBank nucleotide accession number PZ567662) was obtained via RT-PCR cloning. Its open reading frame is 726 bp in length and encodes a putative protein consisting of 241 amino acids. Given the lack of 5′ and 3′ transcript end confirmation, this sequence may represent a partial CDS. InterProScan analysis suggested that the protein belongs to the β-lactamase superfamily (Lactamase_B, PF00753, residues 56–224) and contains a POD-like_MBL-fold domain (Persulfide dioxygenase-like, MBL-fold metallo-hydrolase domain; cd07724, residues 46–226). Iron-binding sites are located at residues 102, 158, and 183, and active sites involve residues 102, 106, 158, 183, 186, 190, 191, 192, and 226 (Figure 1B).

The physicochemical properties of JcETHE1 and its homologues from *Arabidopsis*, rice, and grapevine were predicted and compared (Table 1). JcETHE1 consists of 241 amino acids, corresponding to a molecular mass of 26.53 kDa and a theoretical pI of 8.86. The instability index, which estimates protein stability in vitro, suggests an unstable protein when the value exceeds 40. All five ETHE1 homologues exhibited instability indices well below this threshold (JcETHE1, 25.44; AtETHE1, 36.58; OsETHE1, 37.54; VvETHE1A, 26.35; VvETHE1B, 29.01), and were therefore predicted to be stable. The aliphatic index, a measure of relative thermostability, was highest for JcETHE1 (95.52), followed by OsETHE1 (91.15) and the grapevine homologues (88.67 and 86.67), with AtETHE1 having the lowest value (85.51). Grand average of hydropathy (GRAVY) values were negative for all homologues (−0.222 to −0.032), indicating a hydrophilic character. Subcellular localization prediction consistently assigned each ETHE1 protein to the mitochondrion, which is consistent with the predicted role of these proteins in mitochondrial sulfide detoxification.

### 3.2. Phylogenetic Relationship and Conserved Motif Analyses of JcETHE1

**Figure 2:**
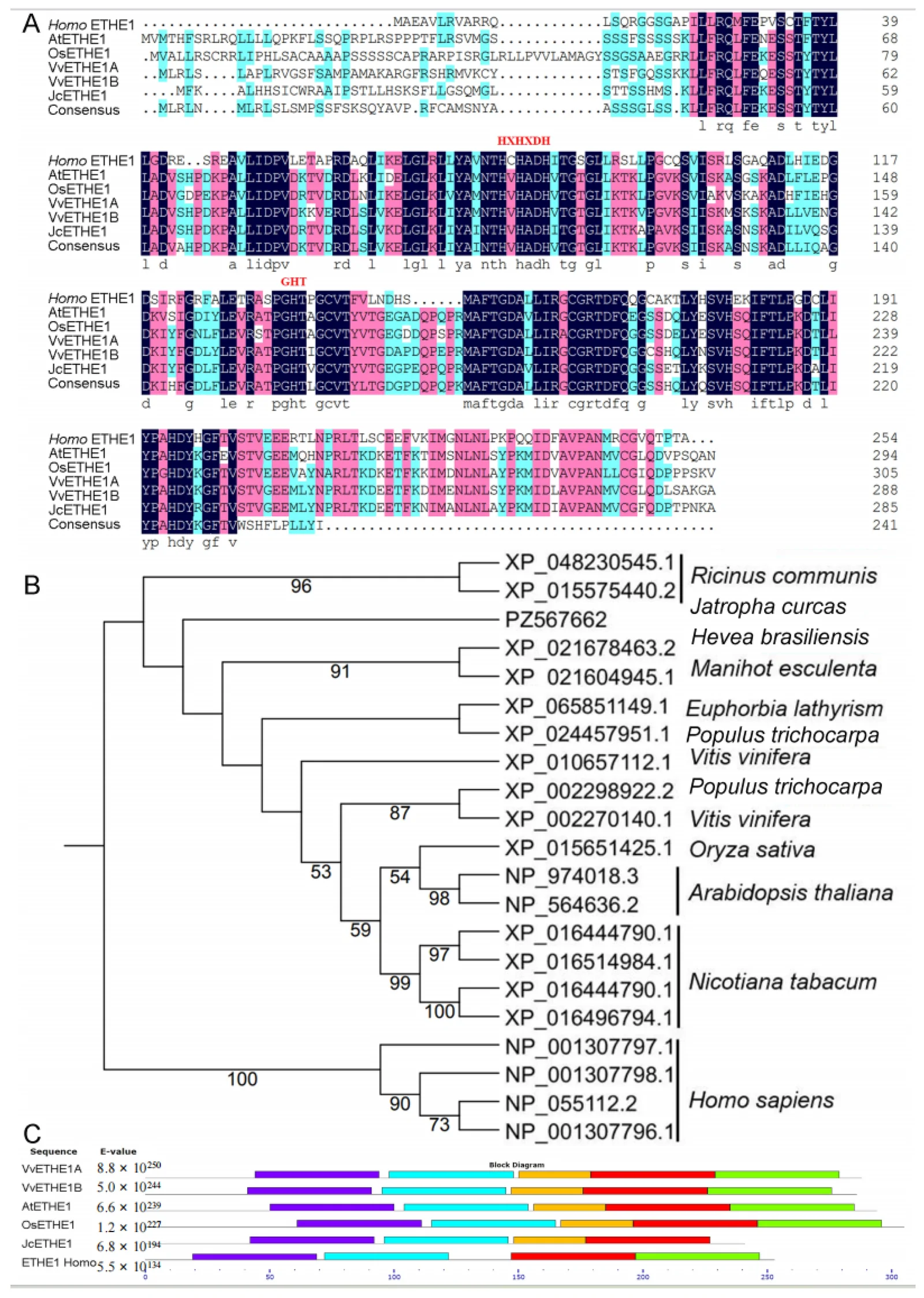
Amino acid sequence alignment (**A**), phylogenetic analysis (**B**) of ETHE1 homologues, and conserved motif identification using MEME analysis (**C**). A: the background shading indicates amino acid conservation. Dark backgrounds denote strictly conserved residues (100% identity), while colored backgrounds represent residues with similar physicochemical properties. Unshaded regions indicate non-conserved positions. C: different colored boxes represent distinct motifs: red, Motif 1; blue, Motif 2; green, Motif 3; purple, Motif 4; orange, Motif 5 (see Table 2). Note that to display intraspecific sequence variance, multiple isoforms from the same species were retained in the dataset. The resulting redundancy may contribute to the low bootstrap support observed at some internal basal nodes. The nucleotide sequence reported in this study is available under GenBank accession no. PZ567662. The corresponding protein accession number will be automatically assigned by NCBI upon final processing of the submission.

The amino acid sequence of JcETHE1 was used as a query in the NCBI Protein BLAST (version 2.17.0) online sequence analysis tool. The results suggested that JcETHE1 exhibits high homology with ETHE1 amino acid sequences from other species. Multiple sequence alignment performed using DNAMAN revealed the presence of the HXHXDH metal-binding motif (represented by the HVHADH sequence in JcETHE1) and the GHT substrate-binding site in the ETHE1 protein sequences of all species examined (Figure 2A).

To clarify the phylogenetic relationships between JcETHE1 and its homologues from other species, an ML phylogenetic tree was constructed based on the amino acid alignment of 21 sequences (Figure 2B). The tree revealed a clear divergence between plant and animal ETHE1 proteins, with the plant clade receiving robust support (100% bootstrap) when rooted with the human ETHE1 sequences as the out-group. Within the plant clade, JcETHE1 specifically clustered with ETHE1 proteins from *Hevea brasiliensis* and *Manihot esculenta* with strong support (91% bootstrap), forming a small subclade. This subclade further grouped with homologues from *Ricinus communis*, and together they clustered with sequences from *Euphorbia lathyris* and *Populus trichocarpa*. These clusters further grouped with homologues from *Vitis vinifera*, although the support for this broader grouping was relatively low, suggesting that the branching order within this large angiosperm clade is not strongly resolved. One contributing factor to this weak resolution is that we deliberately retained multiple isoforms from the same species (e.g., four *Nicotiana tabacum* isoforms and two *R. communis* isoforms) in the dataset to illustrate the sequence variance occurring within species. Nevertheless, we acknowledge that this inclusion of redundant homologues may introduce noise, further contributing to the low statistical support observed at several internal nodes. Consequently, while the distinct separation between plant and animal ETHE1 clades is well-supported, the precise branching order within the higher plant subclades, including the specific placement of JcETHE1 relative to other dicot homologues, remains largely unresolved and should be interpreted with caution.

Motif analysis identified a total of five conserved motifs (Motifs 1–5), with lengths of 50, 50, 50, 50, and 29 amino acids, respectively (Table 2). However, only Motifs 1, 2, 4, and 5 were detected in the JcETHE1 sequence, while Motif 3 was absent (Figure 2C). All detected motifs in JcETHE1 were highly conserved, with a MAST search E-value of 6.8 × 10^−194^ (Figure 2C). The variation in protein length among ETHE1 homologues is predominantly attributed to species-specific insertions/deletions in the non-conserved N- and C-terminal extensions. Despite the differences in protein length, the predicted JcETHE1 sequence retains several conserved residues and motifs associated with the putative catalytic domain. However, the structural and functional completeness of this domain remains to be experimentally confirmed. However, the absence of Motif 3 in the JcETHE1 sequence suggests that the structural completeness of its catalytic domain cannot be fully confirmed.

### 3.3. Predicted Secondary and Tertiary Structure of JcETHE1

**Figure 3:**
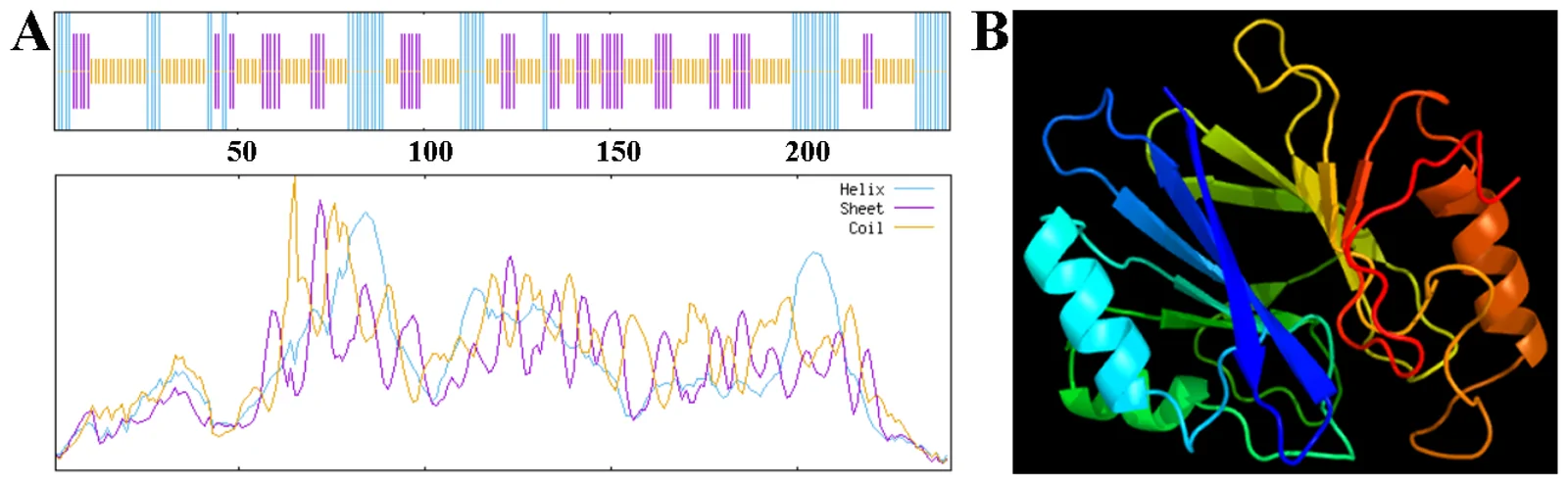
Prediction of secondary (**A**) and tertiary (**B**) structure of JcETHE1 protein. Note: Blue, α-helices; purple, extended strands; green, β-sheets; yellow, random coils (**A**).

The results of secondary structure analysis of the JcETHE1 protein showed that the protein is mainly composed of random coils (53.53%), followed by extended strands (23.65%) and alpha-helices (22.82%), as shown in Figure 3A. InterPro search and QuickGO analysis revealed that JcETHE1 is predicted to possess sulfur dioxygenase activity (GO:0050313), is predicted to participate in the glutathione metabolic process (GO:0006749) and the hydrogen sulfide metabolic process (GO:0070813), and is predicted to localize to the mitochondrion (GO:0005739). The tertiary structure of JcETHE1 was predicted using Phyre2. Figure 3B shows the tertiary structure model of residues 42–238 of JcETHE1. The model was built using the template c2yuaA with a sequence identity of 36% and a coverage of 196 out of 241 residues (~81%). The confidence score of this prediction was 100%, which represents the confidence of the template match, although it does not guarantee absolute structural accuracy. The remaining amino acids are regions not modeled by Phyre2, likely due to the limited coverage of the selected template.

### 3.4. cis-Regulatory Elements in the Promoter Regions of JcETHE1

**Figure 4:**
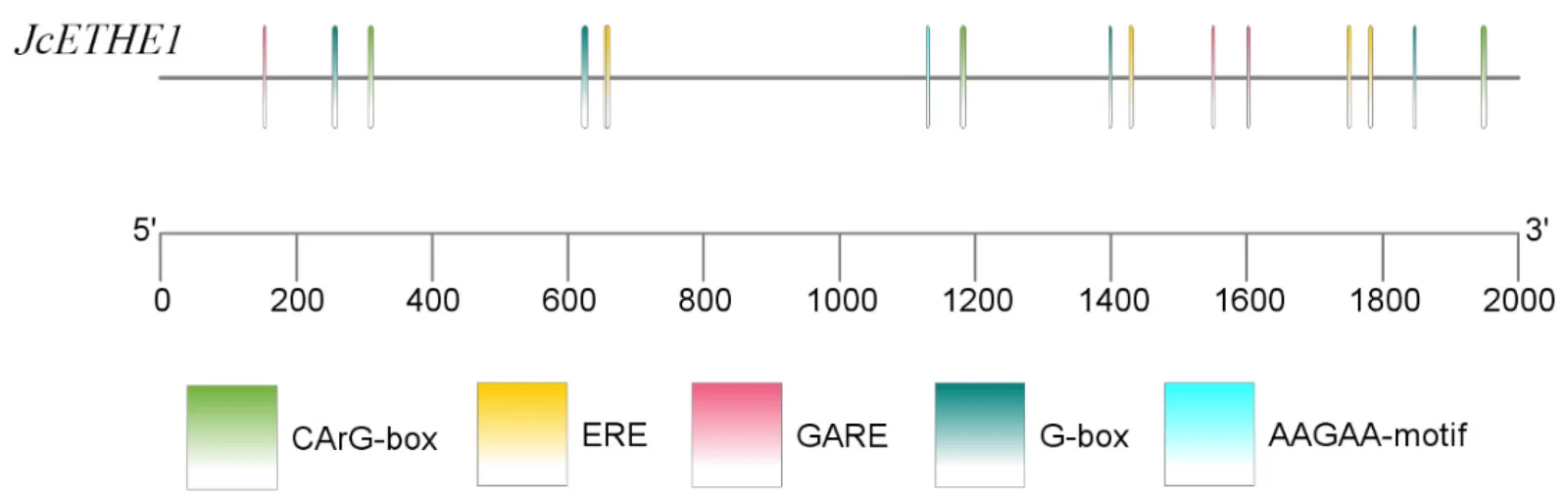
Analysis of in silico predicted *cis*-regulatory elements associated with flower development and hormone signaling in the *JcETHE1* gene promoter. Note: The figure shows the distribution of predicted elements. The functional activity of these motifs requires experimental validation.

A promoter is a DNA sequence located upstream of the 5′ untranslated region of a gene that achieves precise spatiotemporal control of gene expression through the specific binding of RNA polymerase and various transcription factors [32]. Promoter regions typically consist of core elements such as the TATA-box and CAAT-box, as well as a diverse array of *cis*-regulatory elements, including light-responsive elements and hormone-responsive elements, among other transcriptional regulatory sequences. Collectively, these elements enable genes to respond to endogenous signaling molecules (e.g., ABA, jasmonic acid) and external environmental factors (e.g., light, drought stress) [33,34]. To investigate the potential transcriptional framework governing JcETHE1 in *J. curcas*, we analyzed the 2000 bp segment directly upstream of its initiation codon using both the PlantCARE and PlantPAN 4.0 databases. A total of 143 putative *cis*-regulatory elements were predicted, of which 41 functional elements were identified after excluding core promoter motifs (TATA-box and CAAT-box) (Table 3). These predicted elements were categorized into several groups: light-responsive, hormone-responsive, stress-responsive, and development-related elements (Figure 4). The detailed information including the exact sequence, position, strand, and the source database for each predicted element is provided in Table 4.

Further analysis revealed that the promoter sequence also contains key elements associated with flower development and hormone signaling. Among the hormone-responsive elements, four ABA-responsive elements (ABRE/ABRE3a/ABRE4), five ethylene-responsive elements (EREs), three gibberellin-responsive elements (GAREs), and four G-boxes were detected. Among the flower development-related elements, three CArG-boxes (MADS-box transcription factor binding sites) and one pollen/stamen-specific element AAGAA-motif were identified (Figure 4 and Table 3). Additionally, the promoter contains multiple stress-responsive elements (ARE, STRE, WRE3, MYB, MYC) and two endosperm-specific elements (GCN4_motif) (Table 3).

## 4. Discussion

*J. curcas* is an economically important energy plant known for its high seed oil content, making it a promising feedstock for biodiesel [3]. However, its seed yield is severely limited by the extremely low proportion of female flowers within the inflorescence and by its frequent exposure to abiotic stresses such as drought, high temperature, and salinity in marginal cultivation areas [7,35]. These constraints highlight the need to identify key genes that simultaneously regulate flower development and stress responses for molecular breeding. The *ETHE1* gene encodes a mitochondrial sulfur dioxygenase that catalyzes the oxidation of glutathione persulfide (GSSH) to sulfite, a critical step in the detoxification of sulfide generated during cysteine catabolism [9,13,36,37]. In plants, this pathway not only maintains sulfide homeostasis but also links amino acid turnover to central metabolism and stress adaptation [8,13,14,15]. Therefore, investigating ETHE1 in *J. curcas* provides an opportunity to understand how sulfur metabolism intersects with reproductive development and environmental resilience in an oilseed crop.

Promoter *cis*-regulatory element prediction revealed that the *JcETHE1* promoter contains multiple putative functional elements associated with light response, hormone responses, stress, and endosperm/seed development. Among them, putative elements such as G-box/ABRE, MYB/MYC, and ARE coexist in the promoter region (Table 3), suggesting that the gene has the potential to achieve fine transcriptional regulation by integrating multiple signals. This phenomenon is similar to the synergistic action of DRE and ABRE elements in the *Arabidopsis* rd29A promoter [38,39]. In rice, the promoter activity of *OsETHE1* is regulated by light signals and calcium, and stress responses are suppressed under dark conditions [8]. Therefore, the predicted G-box and ABRE elements in the *JcETHE1* promoter may potentially mediate the cross-regulation between ABA and light signals. It has been experimentally demonstrated that *AtETHE1* is highly expressed in the endosperm [12]. Consistently, the presence of the GCN4_motif in the *JcETHE1* promoter raises the hypothesis that this gene may be involved in seed development in *J. curcas*, pending future experimental validation.

In addition, CArG-box, GARE (gibberellin-responsive element), and ERE (ethylene-responsive element) simultaneously exist in the promoter (Table 3 and Figure 4). CArG-box is a typical binding site for MADS-box transcription factors; MADS-domain transcription factors bind to double-stranded DNA as dimers and recognize the CArG-box core sequence (e.g., 5′-CC(A/T)6GG-3′), playing a central role in floral organ identity determination [40]. In *Arabidopsis*, MADS-box proteins such as APETALA3 directly regulate petal and stamen development by binding to CArG-box [40,41]. GARE mediates gibberellin signaling [42] and participates in stamen development [43], whereas ERE responds to ethylene signaling and is associated with stamen abortion in female flowers [44]. Furthermore, the AAGAA-motif, which is reported to function as a pollen/stamen-specific expression element, further supports the potential function of this gene in stamen tissues [45]. However, it must be emphasized that the co-existence of CArG-box, GARE, and ERE suggests that JcETHE1 may potentially be regulated at the intersection of gibberellin and ethylene signaling pathways and may potentially be targeted by MADS-box transcription factors, potentially influencing the fate of stamen development or abortion. This proposed regulation remains purely predictive. This regulatory architecture is particularly relevant to *J. curcas*, where an improved understanding of floral organ development could help overcome the low female-to-male flower ratio. Moreover, the presence of multiple stress-responsive and ABA-related elements suggests that JcETHE1 may be poised to respond to drought and other abiotic challenges, which is critical for a crop commonly cultivated in hot, dry environments.

Functional studies in other plant species have shown that ETHE1 exhibits tissue-specific expression patterns. For instance, the flower-predominant expression pattern of *AtETHE1* in *Arabidopsis* inflorescences [12] and its strong immunolocalization signal in tapetal cells [12] imply a conserved role for ETHE1 in reproductive development. Given that sulfide detoxification is essential for sustaining high metabolic activity in rapidly developing organs, the potential expression of *JcETHE1* in flowers may reflect an increased demand for sulfur detoxification during floral organogenesis. However, rice *OsETHE1* is specifically expressed in roots [8], suggesting functional divergence between monocots and dicots, as well as between woody and herbaceous plants. The specific expression profile of *JcETHE1* in *J. curcas* requires future experimental validation.

While the divergence between monocots (rice) and dicots (*J. curcas*) provides an evolutionary context for the expression differences observed in other ETHE1 homologues, the high metabolic demand associated with *Jatropha’s* complex inflorescences offers a complementary physiological explanation. *J. curcas* produces highly complex unisexual inflorescences with a high male-to-female flower ratio, which requires intense cell division and energy-consuming biosynthetic processes during floral organogenesis. Given that sulfide detoxification via ETHE1 is essential for maintaining high metabolic activity in rapidly developing organs, the potential elevated expression of *JcETHE1* in flowers (as hypothesized from our promoter analysis and inferred from other ETHE1 studies) may reflect an increased demand for sulfur detoxification to protect the actively dividing floral tissues from sulfide toxicity. This physiological requirement, coupled with the specific metabolic architecture of *J. curcas* inflorescences, likely contributes to a putative tissue predominance, rather than it being solely a consequence of monocot-dicot evolutionary divergence.

The ability of ETHE1 to participate in both developmental processes and stress responses raises the possibility that modulating *JcETHE1* expression could potentially simultaneously improve flower development and stress tolerance, thereby offering a theoretical avenue for enhancing seed yield under adverse environments.

### Limitations of the Study

It should be explicitly acknowledged that the current study is a preliminary molecular characterization based predominantly on in silico analyses and sequence homology, along with the initial molecular cloning of the JcETHE1 coding sequence. Furthermore, as the cloned *JcETHE1* coding sequence encodes a shorter protein (241 aa) compared to its homologues and lacks Motif 3, its completeness cannot be guaranteed without experimental confirmation of the 5′ and 3′ transcript ends. The claims regarding the potential function of *JcETHE1* are purely predictive. No enzymatic activity assay, heterologous complementation, transgenic (overexpression/knockdown/CRISPR) analysis, or promoter–reporter (e.g., GUS/luciferase) assay was performed in this study. Therefore, the putative catalytic residues, the potential roles in flower development and stress responses, and the proposed regulation by gibberellin and ethylene signaling should not be interpreted as demonstrated mechanisms, but rather as testable hypotheses. To move beyond these predictions, future research should focus on: (i) 5′ and 3′ Rapid Amplification of cDNA Ends (RACE) to verify the complete transcript boundaries and rule out possible alternative transcripts or annotation errors; (ii) recombinant protein expression and enzymatic activity assays to confirm sulfur dioxygenase function; (iii) promoter–GUS analysis under various hormone (ABA, GA, ethylene) and stress treatments to validate promoter function; (iv) background-promoter enrichment analysis against a random promoter set and basic tissue/stress qRT-PCR surveys to substantiate any functional claims; (v) generating *JcETHE1* overexpression or RNAi knockdown lines to evaluate its physiological roles *in planta*; (vi) quantitative RT-PCR analysis following exogenous ABA, drought, salt, or temperature treatments to validate the predicted stress-responsiveness of its promoter; (vii) transient expression assays in plant cells (e.g., Nicotiana *benthamiana* leaves or onion epidermal cells) using a *JcETHE1*-GFP fusion construct to confirm the predicted mitochondrial subcellular localization; and (viii) detailed expression profiling of *JcETHE1* during different developmental stages of seed maturation (e.g., early, mid, and late stages) to clarify whether this gene functions primarily in floral organogenesis or plays a role in seed maturation. Until these experiments are conducted, *JcETHE1* should be considered a candidate gene for further functional studies, rather than a validated molecular breeding target.

## 5. Conclusions

*J. curcas* is an important energy crop characterized by high seed oil content, yet its productivity is limited by an extremely low female-to-male flower ratio and susceptibility to abiotic stresses. In this study, we cloned a putative *JcETHE1* coding sequence, to our knowledge, for the first time and systematically characterized its structural features, conserved motifs, phylogenetic relationships, and promoter *cis*-regulatory elements. Because the cloned sequence (241 aa) lacks Motif 3 and its transcript ends were not experimentally confirmed, it may potentially represent a partial CDS. The encoded protein is a putative mitochondrion-localized sulfur dioxygenase belonging to the metallo-β-lactamase superfamily, possessing conserved iron-binding sites and the characteristic HVHADH and GHT motifs, indicating the presence of conserved putative catalytic domains for sulfide detoxification. However, enzymatic activity, subcellular localization, and the structural completeness of the protein require experimental validation. Promoter analysis identified key putative regulatory elements associated with flower development (CArG-box, GARE, ERE, AAGAA-motif), hormone signaling (ABA, ethylene, gibberellin), and stress responses (MYB, MYC, ARE), suggesting a potential, yet unvalidated, role for *JcETHE1* in coordinating reproductive development and environmental adaptation. These results provide a molecular basis for future studies aimed at understanding how sulfur metabolism intersects with flower development and stress tolerance in *J. curcas*. The co-occurrence of floral and stress-related regulatory elements in the promoter supports the designation of *JcETHE1* as a candidate gene for future functional studies aimed at improving our understanding of reproductive development and stress resilience in *J. curcas*. Before it can be considered a molecular breeding target, its biological roles must be confirmed through experimental validation (e.g., gene overexpression/knockout and stress treatments).

## Figures and Tables

**Table 1:** Predicted characteristics of ETHE1 proteins in *Arabidopsis*, rice, *J. curcas*, and grapevine.

Accession Number	Protein Name	AA	Predicted Molecular Weight (kDa)	Predicted Theoretical PI	Predicted Instability Index	Predicted Aliphatic Index	Predicted GRAVY	Predicted Subcellular Location
NP_564636.2	AtETHE1	294	32.33	6.5	36.58	85.51	−0.222	Mitochondrion
XP_015651425	OsETHE1	304	33.17	8.14	37.54	91.15	−0.120	Mitochondrion
PZ567662 (Nucleotide Accession; Predicted Translation)	JcETHE1	241	26.53	8.86	25.44	95.52	−0.032	Mitochondrion
XP_010657112	VvETHE1A	288	31.86	8.46	26.35	86.67	−0.177	Mitochondrion
XP_002270140	VvETHE1B	286	31.43	7.7	29.01	88.67	−0.101	Mitochondrion

Note: The *JcETHE1* entry (PZ567662) represents a nucleotide accession number. The amino acid sequence listed is the predicted translation of its cloned CDS. The corresponding genomic locus is located on the reverse (complement) strand at positions 1,343,323–1,354,051 of the scaffold NW_023591682.1 (Genome Assembly ID: GCF_014843425.1). The official protein accession number will be automatically assigned by NCBI upon final processing of the submission.

**Table 2:** Conserved motifs identified in the JcETHE1 protein.

Motif	Length (aa)	Representative Sequence	Conserved Feature
1	50	MAFTGDALLIRGCGRTDFQGGSSDQLYESVHSQIFTLPKDTLIYPAHDYK	Fe binding site
2	50	YAMNTHVHADHVTGTGLIKTKLPGVKSIISKASGSKADLLIENGDKIYFG	HVHADH metal-binding motif
3	50	GFTVSTVGEEMLYNPRLTKDKETFKKIMENLNLAYPKMIDVAVPANMVCG	-
4	50	KLLFRQLFEKESSTYTYLLADVSHPDKPALLIDPVDKTVDRDLSLVKELG	-
5	29	FLEVRATPGHTAGCVTYVTGEGPDQPQPR	GHT substrate-binding site

Note: Conserved features in the representative sequences are shown in red.

**Table 3:** Functional classification of *cis*-regulatory elements in the *JcETHE1* promoter.

Functional Category	Element Name	Number	Functional Description
Core promoter elements	TATA-box	58	RNA polymerase II binding site; involved in transcription initiation
	CAAT-box	43	Enhances transcription efficiency; widely present in promoter regions
	AT~TATA-box	1	Similar to TATA-box; may participate in basal transcription
Light-responsive elements	G-Box	4	Involved in light response; regulated by ABA and environmental stress
	GATA-motif	1	Part of light-responsive elements
	GA-motif	1	Light-response-related element
	TCT-motif	1	Light-response-related element
Hormone-responsive elements	ABRE/ABRE3a/ABRE4	4	ABA-responsive elements; involved in drought, salt, and low-temperature stress responses
	ERE	5	Ethylene-responsive elements; regulate development and stress responses
	GARE	3	Gibberellin-responsive elements; mediate gibberellin signal transduction and regulate downstream gene expression essential for stamen development
Flower development-related elements	CArG-box	3	MADS-box transcription factor binding sites; core elements for floral organ identity determination (ABC model); involved in precise regulation of stamen and carpel development
	AAGAA-motif	1	Pollen/stamen-specific expression regulation; acts as an enhancer element to activate downstream gene expression specifically in pollen
Stress-responsive elements	ARE	1	*cis*-acting regulatory element essential for anaerobic induction
	STRE	1	General stress-responsive element; responds to heat and osmotic stress
	WRE3	1	Involved in biotic/abiotic stress; wound-inducible
	MYB	2	MYB binding sites; involved in drought and salt stress regulation
	MYC	4	MYC binding sites; involved in stress and hormone signaling
	MYB-like sequence	1	MYB-like binding site; involved in transcriptional regulation
Endosperm/seed development elements	GCN4_motif	2	Endosperm-specific expression elements
Others	AT-rich element	1	Binding site for AT-rich DNA-binding proteins
Unknown function	Unnamed_4	5	Elements with unknown function

**Table 4:** Detailed information of the predicted *cis*-regulatory elements and their physical locations in the *JcETHE1* promoter.

Functional Category	Element Name	Motif Sequence (5′→3′)	Position (Start–End)	Strand	Source Database
Hormone-responsive	ABRE	ACGTG	1410–1414	−	PlantCARE
	ABRE	ACGTG	1859–1863	−	Both (PlantCARE and PlantPAN 4.0)
	ABRE3a	TACGTG	1410–1415	−	PlantCARE
	ABRE4	CACGTA	1410–1415	+	Both
	ERE	ATTTTAAA	667–674	+	Both
	ERE	ATTTTAAA	669–676	−	Both
	ERE	ATTTTAAA	1440–1447	+	PlantCARE
	ERE	ATTTTAAA	1761–1768	−	Both
	ERE	ATTTTAAA	1793–1800	−	PlantCARE
	GARE	AAACAGA	411–417	+	PlantCARE
	GARE	AAACAGA	462–468	+	PlantCARE
	GARE	AAACAGA	1172–1178	−	PlantCARE
Light-responsive	G-Box	TCCACATGGCA	634–644	−	Both
	G-Box	GCCACGTGGA	266–275	+	Both
	G-Box	CACGTT	1859–1864	+	Both
	G-box	TACGTG	1410–1415	−	Both
	GA-motif	ATAGATAA	121–128	−	Both
	GATA-motif	AAGGATAAGG	854–863	+	PlantCARE
	TCT-motif	TCTTAC	576–581	−	PlantCARE
Stress-responsive	ARE	AAACCA	1562–1567	+	Both
	STRE	AGGGG	1229–1233	+	PlantCARE
	WRE3	CCACCT	269–274	−	Both
	MYB	CAACCA	224–229	+	PlantCARE
	MYB	TAACCA	1592–1597	−	Both
	MYB-like	TAACCA	1592–1597	−	PlantCARE
	MYC	CAATTG	228–233	+	Both
	MYC	CATGTG	637–642	+	Both
	MYC	CATTTG	760–765	−	Both
	MYC	CATTTG	932–937	−	PlantCARE
Flower development	AAGAA-motif	GAAAGAA	1141–1147	+	PlantCARE
	CArG-box	CCATTTTTGG	876–885	+	PlantCARE
	CArG-box	CCTTTTTTG	1531–1540	−	PlantCARE
	CArG-box	CCTTTTTTG	1140–1149	+	PlantCARE
Endosperm development	GCN4_motif	TGAGTCA	650–656	+	Both
	GCN4_motif	TGAGTCA	1101–1107	−	PlantCARE
Others	AT-rich element	ATAGAAATCAA	500–510	−	PlantCARE

Note: The databases used (PlantCARE and PlantPAN 4.0) identify elements based on consensus sequences and do not provide a directly comparable quantitative matrix score. Therefore, only the motif sequence, physical position, strand, and source database are reported. In the “Strand” column, “+” indicates that the motif is located on the forward (sense) strand of the DNA sequence, whereas “−” indicates that the motif is located on the reverse (anti-sense) strand.

## Data Availability

The data presented in this study are openly available. The cloned nucleotide sequence of *JcETHE1* has been deposited in the NCBI GenBank database under the accession number PZ567662 (released immediately for public access). The translated protein sequence, 2000-bp upstream promoter sequence, list of accession numbers, multiple sequence alignment, and the phylogenetic tree are provided as Supplementary Materials Table S1, Table S2, File S1, and File S2, respectively.

## Supplementary Materials

The following supporting information can be downloaded at: https://www.mdpi.com/article/10.3390/cimb48090919/s1.

## Abbreviations

The following abbreviations are used in this manuscript:

*ETHE1*	sulfur dioxygenase
*J. curcas*	*Jatropha curcas*
ABA	Abscisic Acid
ABREs	ABA-responsive elements
EREs	ethylene-responsive elements
GAREs	gibberellin-responsive elements

## References

[1] Al-Khayri J.M., Sudheer W.N., Preetha T.R., Nagella P., Rezk A.A., Shehata W.F. (2022). Biotechnological research progress in *Jatropha*, a biodiesel-yielding plant. Plants.

[2] Fendiyanto M.H., Anshori M.F., Pratami M.P., Wasonga D.O., Seleiman M.F. (2024). Metabolite comparative variation related lipid metabolisms among fruit, leaf, and stem of *Jatropha curcas*. Heliyon.

[3] Cao X., Wei X., Shao Y., Li D., Zhu J. (2025). *Jatropha curcas* seed oil for possible human consumption: A toxicological assessment of its phorbol esters. Toxicol. Rep..

[4] Lei S., Zhao L., Chen Y., Xu G. (2023). Identification and promoter analysis of a GA-responsive gene in *Jatropha curcas*. Plant Cell Rep..

[5] Wang J., Bai X., Su Y., Deng H., Cai L., Ming X., Tao Y.-B., He H., Xu Z.-F., Tang M. (2023). *JCSEUSS1* negatively regulates reproductive organ development in perennial woody *Jatropha curcas*. Planta.

[6] Silva-Mann R., dos Santos Pessoa A.M., Ribeiro D.O., Ferreira O.J.M., Rabbani A.R.C., Nunes V.V., Calazans C.C., de Lima Nogueira P.C. (2023). Parental selection and the diversity of the F1 *Jatropha curcas* genotypes: Seed quality and phytochemistry. Ind. Crops Prod..

[7] Chen Y., Wang H., Chen K., Cui M., Gong M. (2013). The research progress on stress resistance of energy plant *Jatropha curcas* L.. Chin. Agric. Sci. Bull..

[8] Kaur C., Mustafiz A., Sarkar A.K., Ariyadasa T.U., Singla-Pareek S.L., Sopory S.K. (2014). Expression of abiotic stress inducible ETHE1-like protein from rice is higher in roots and is regulated by calcium. Physiol. Plant..

[9] Zhang L. (2015). Advances of sulfur dioxygenase—A key enzyme in the mitochondrial sulfide oxidation. Jining Med. Univ..

[10] Yan J., Song J.-N., Wang L., Wang Y.-J., Zhang C.-H. (2020). Cloning and expression analysis of sulfur dioxygenase gene *VvETHE1* in grapevine (*Vitis vinifera*). J. Agric. Biotechnol..

[11] Tiranti V., D’Adamo P., Briem E., Ferrari G., Mineri R., Lamantea E., Mandel H., Balestri P., Garcia-Silva M., Vollmer B. (2004). Ethylmalonic encephalopathy is caused by mutations in *ETHE1*, a gene encoding a mitochondrial matrix protein. Am. J. Hum. Genet..

[12] Holdorf M.M., Owen H.A., Lieber S.R., Yuan L., Adams N., Dabney-Smith C., Makaroff C.A. (2012). *Arabidopsis* ETHE1 encodes a sulfur dioxygenase that is essential for embryo and endosperm development. Plant Physiol..

[13] Krüßel L., Junemann J., Wirtz M., Birke H., Thornton J.D., Browning L.W., Poschet G., Hell R., Balk J., Braun H.-P. (2014). The mitochondrial sulfur dioxygenase ETHYLMALONIC ENCEPHALOPATHY PROTEIN1 is required for amino acid catabolism during carbohydrate starvation and embryo development in *Arabidopsis*. Plant Physiol..

[14] Lorenz C., Brandt S., Borisjuk L., Rolletschek H., Heinzel N., Tohge T., Fernie A.R., Braun H.-P., Hildebrandt T.M. (2018). The role of persulfide metabolism during *Arabidopsis* seed development under light and dark conditions. Front. Plant Sci..

[15] Kaur C., Singla-Pareek S.L., Sopory S.K. (2014). Stress response of *OsETHE1* is altered in response to light and dark conditions. Plant Signal. Behav..

[16] Jalali S., Kancharla N., Yepuri V., Arockiasamy S. (2020). Exploitation of Hi-C sequencing for improvement of genome assembly and in-vitro validation of differentially expressing genes in *Jatropha curcas* L.. 3 Biotech.

[17] Hunter S., Apweiler R., Attwood T.K., Bairoch A., Bateman A., Binns D., Bork P., Das U., Daugherty L., Duquenne L. (2009). InterPro: The integrative protein signature database. Nucleic Acids Res..

[18] Gasteiger E., Hoogland C., Gattiker A., Duvaud S., Wilkins M.R., Appel R.D., Bairoch A., Walker J.M. (2005). Protein identification and analysis tools on the ExPASy server. The Proteomics Protocols Handbook.

[19] Chou K.C., Shen H.B. (2010). Plant-mPLoc: A top-down strategy to augment the power for predicting plant protein subcellular localization. PLoS ONE.

[20] Geourjon C., Deléage G. (1995). SOPMA: Significant improvements in protein secondary structure prediction by consensus prediction from multiple alignments. Bioinformatics.

[21] Kelley L.A., Mezulis S., Yates C.M., Wass M.N., Sternberg M.J.E. (2015). The Phyre2 web portal for protein modeling, prediction and analysis. Nat. Protoc..

[22] Powell H.R., Islam S.A., David A., Sternberg M.J.E. (2025). Phyre2.2: A community resource for template-based protein structure prediction. J. Mol. Biol..

[23] Bailey T.L., Johnson J., Grant C.E., Noble W.S. (2015). The MEME suite. Nucleic Acids Res..

[24] Bailey T.L., Gribskov M. (1998). Combining evidence using p-values: Application to sequence homology searches. Bioinformatics.

[25] Lescot M., Déhais P., Thijs G., Marchal K., Moreau Y., Van de Peer Y., Rouzé P., Rombauts S. (2002). PlantCARE, a database of plant cis-acting regulatory elements and a portal to tools for in silico analysis of promoter sequences. Nucleic Acids Res..

[26] Chow C.N., Yang C.W., Wu N.Y., Wang H.T., Tseng K.C., Chiu Y.H., Lee T.Y., Chang W.C. (2024). PlantPAN 4.0: Updated database for identifying conserved non-coding sequences and exploring dynamic transcriptional regulation in plant promoters. Nucleic Acids Res..

[27] Lamesch P., Berardini T.Z., Li D., Swarbreck D., Wilks C., Sasidharan R., Muller R., Dreher K., Alexander D.L., Garcia-Hernandez M. (2012). The *Arabidopsis* Information Resource (TAIR): Improved gene annotation and new tools. Nucleic Acids Res..

[28] Edgar R.C. (2022). Muscle5: High-accuracy alignment ensembles enable unbiased assessments of sequence homology and phylogeny. Nat. Commun..

[29] Kalyaanamoorthy S., Minh B.Q., Wong T.K.F., von Haeseler A., Jermiin L.S. (2017). ModelFinder: Fast model selection for accurate phylogenetic estimates. Nat. Methods.

[30] Nguyen L.-T., Schmidt H.A., von Haeseler A., Minh B.Q. (2015). IQ-TREE: A fast and effective stochastic algorithm for estimating maximum-likelihood phylogenies. Mol. Biol. Evol..

[31] Minh B.Q., Nguyen M.A.T., von Haeseler A. (2013). Ultrafast approximation for phylogenetic bootstrap. Mol. Biol. Evol..

[32] Lenhard B., Sandelin A., Carninci P. (2012). Metazoan promoters: Emerging characteristics and insights into transcriptional regulation. Nat. Rev. Genet..

[33] Priest H.D., Filichkin S.A., Mockler T.C. (2009). *cis*-Regulatory elements in plant cell signaling. Curr. Opin. Plant Biol..

[34] Hernandez-Garcia C.M., Finer J.J. (2014). Identification and validation of promoters and cis-acting regulatory elements. Plant Sci..

[35] Hui W., Yang Y., Wu G., Peng C., Chen X., Zayed M.Z. (2017). Transcriptome profile analysis reveals the regulation mechanism of floral sex differentiation in *Jatropha curcas* L.. Sci. Rep..

[36] Birke H., Hildebrandt T.M., Wirtz M., Hell R. (2015). Sulfide detoxification in plant mitochondria. Methods Enzymol..

[37] Höfler S., Lorenz C., Busch T., Brinkkötter M., Tohge T., Fernie A.R., Braun H.P., Hildebrandt T.M. (2016). Dealing with the sulfur part of cysteine: Four enzymatic steps degrade l-cysteine to pyruvate and thiosulfate in *Arabidopsis* mitochondria. Physiol. Plant..

[38] Yamaguchi-Shinozaki K., Shinozaki K. (1994). A novel cis-acting element in an Arabidopsis gene is involved in responsiveness to drought, low-temperature, or high-salt stress. Plant Cell.

[39] Narusaka Y., Nakashima K., Shinwari Z., Sakuma Y., Furihata T., Abe H., Narusaka M., Shinozaki K., Yamaguchi-Shinozaki K. (2003). Interaction between two cis-acting elements, ABRE and DRE, in ABA-dependent expression of Arabidopsis rd29A gene in response to dehydration and high-salinity stresses. Plant J..

[40] Käppel S., Rümpler F., Theißen G. (2023). Cracking the floral quartet code: How do multimers of MIKC^C-type MADS-domain transcription factors recognize their target genes?. Int. J. Mol. Sci..

[41] Tilly J., Allen D., Jack T. (1998). The CArG boxes in the promoter of the *Arabidopsis* floral organ identity gene *APETALA3* mediate diverse regulatory effects. Development.

[42] Ritchie S., Gilroy S. (2008). Gibberellins: Regulating genes and germination. New Phytol..

[43] Li Q., Zhang L., Wang Y., Huang X. (2019). The research progress of gibberellin on the regulation of flowering and floral organ development in plant. Chin. J. Cell Biol..

[44] Takada K., Ishimaru K., Minamisawa K., Kamada H., Ezura H. (2005). Expression of a mutated melon ethylene receptor gene Cm-ETR1/H69A affects stamen development in *Nicotiana tabacum*. Plant Sci..

[45] Nevosád L., Klodová B., Rudolf J., Raček T., Přerovská T., Kusová A., Svobodová R., Honys D., Procházková Schrumpfová P. (2025). GOLEM: A tool for visualizing the distribution of Gene regulatOry eLEMents within the plant promoters with a focus on male gametophyte. Plant J..

